# *Pseudomonas putida* as a potential biocontrol agent against *Salmonella* Java biofilm formation in the drinking water system of broiler houses

**DOI:** 10.1186/s12866-020-02046-5

**Published:** 2020-12-11

**Authors:** Sharon Maes, Koen De Reu, Stephanie Van Weyenberg, Bram Lories, Marc Heyndrickx, Hans Steenackers

**Affiliations:** 1 Flanders Research Institute for Agriculture, Fisheries and Food (ILVO), Technology and Food Science Unit, Brusselsesteenweg 370, 9090 Melle, Belgium; 2 Faculty of Bioscience Engineering, Department of Microbial and Molecular Systems (M2S), Centre of Microbial and Plant Genetics (CMPG), University of Leuven, Kasteelpark Arenberg 20 box 2460, 3001 Leuven, Belgium; 3 Faculty of Veterinary Medicine, Department of Pathology, Bacteriology and Poultry Diseases, Ghent University, Salisburylaan 133, 9820 Merelbeke, Belgium

**Keywords:** Biocontrol, Biofilm model, Drinking water system, *Pseudomonas putida*, *Salmonella* Java, Social interactions

## Abstract

**Background:**

Environmental biofilms can induce attachment and protection of other microorganisms including pathogens, but can also prevent them from invasion and colonization. This opens the possibility for so-called biocontrol strategies, wherein microorganisms are applied to control the presence of other microbes. The potential for both positive and negative interactions between microbes, however, raises the need for in depth characterization of the sociobiology of candidate biocontrol agents (BCAs). The inside of the drinking water system (DWS) of broiler houses is an interesting niche to apply BCAs, because contamination of these systems with pathogens plays an important role in the infection of broiler chickens and consequently humans. In this study, *Pseudomonas putida*, which is part of the natural microbiota in the DWS of broiler houses, was evaluated as BCA against the broiler pathogen *Salmonella* Java.

**Results:**

To study the interaction between these species, an in vitro model was developed simulating biofilm formation in the drinking water system of broilers. Dual-species biofilms of *P. putida* strains P1, P2, and P3 with *S.* Java were characterized by competitive interactions, independent of *P. putida* strain, *S.* Java inoculum density and application order. When equal inocula of *S*. Java and *P. putida* strains P1 or P3 were simultaneously applied, the interaction was characterized by mutual inhibition, whereas *P. putida* strain P2 showed an exploitation of *S*. Java. Lowering the inoculum density of *S*. Java changed the interaction with *P. putida* strain P3 also into an exploitation of *S*. Java. A further increase in *S*. Java inhibition was established by *P. putida* strain P3 forming a mature biofilm before applying *S.* Java.

**Conclusions:**

This study provides the first results showing the potential of *P. putida* as BCA against *S.* Java in the broiler environment. Future work should include more complex microbial communities residing in the DWS, additional *Salmonella* strains as well as chemicals typically used to clean and disinfect the system.

**Supplementary Information:**

The online version contains supplementary material available at 10.1186/s12866-020-02046-5.

## Background

Infections with *Salmonella* frequently occur in broiler chickens [14], leading to animal disease, animal death and large economic losses. Moreover, the consumption of contaminated poultry meat is a major source of human infections with *Salmonella* [3, 14]. Broiler chickens are mainly infected through environmental sources, feed and drinking water [26]. Drinking water quality and the drinking water system (DWS) therefore play an important role in the general health and performance of broiler chickens [42] and consequently also in human health [59].

Bacteria attaching to the inside of DWSs and forming biofilms are the main source of drinking water contaminations [57]. Not only pathogens such as *Salmonella* spp. are capable of forming biofilms on the materials of the poultry DWS (i.e. plastic [60, 73];), but also commensal species such as *Aeromonas* spp., *E. coli*, *Pseudomonas* spp., and *Sphingomonas* spp. were previously described as biofilm-forming organisms in DWSs ([19]; Van Eenige et al. [66]; Liu et al. [36]; Mulamattathil et al., [40]; van der Wielen and Lut, [67]. These commensal biofilms could provide a niche for the attachment and protection of pathogens [9, 63]. However, as bacterial inter-species interactions are mainly competitive [43], it is more likely that the presence of these commensal microorganisms prevent pathogens from attaching and/or forming a biofilm via competitive exclusion [44].

Commensal microorganisms might therefore be applied to control the presence of pathogens in the DWS. The use of living microorganisms to control other living microbes is called biocontrol. This method could be an alternative for the usually performed chemical disinfection which is not environmentally friendly and poses risks for resistance development [20]. Potential of biocontrol agents (BCAs) to reduce the number of unwanted pathogens and other organisms has already been evaluated, leading to mixed results [8, 39, 72].

Biocontrol is based on the naturally occurring competitive interactions exerted by BCAs on the pathogen. Especially when microbial species occupy the same ecological niche, competitive interactions are expected to be dominant [22]. However, microbes can also engage in other types of social interactions [43]. In a more limited number of cases, microbial species were found to cooperate, enhancing each other’s fitness [51, 52]. This potential for both positive and negative interactions between microbes raises the need for in depth characterization of the sociobiology of candidate BCAs. The *cooperation criterion* can be used to distinguish between cooperative and competitive interactions [43], whereas the *biodiversity effect* (consisting of a selection effect and a complementarity effect) provides a useful logic to further characterize the level and nature of competition [38, 49]. Since biofilm-growth capacities of microorganisms strongly depend on several factors, including growth conditions, contact surface and taxonomy [12, 19, 34, 56, 70], assays to evaluate social interactions should be performed under lab controlled conditions that mimic the real situation as much as possible.

In previous work [41], we identified *Pseudomonas putida* as part of the natural dominant microbiota on several locations on the inside of the DWS in broiler houses, but it is not known as a common contaminant on chickens. Several *Pseudomonas* spp. have been shown to suppress plant pathogens by antibiotic production and more specifically *P. putida* is suggested as a BCA against plant diseases [6, 62]. In the current study, we aimed to investigate whether *P. putida* strains could also serve as BCA against *Salmonella* on the inside of the DWS in broiler houses. We specifically focused on *Salmonella enterica subsp. enterica* serotype Paratyphi B variant Java (hereafter abbreviated as *S.* Java), a serotype that is emerging in Belgian broiler houses [18] and spreads and persists easily in the farm [64, 68]. The interaction between these species was investigated based on the *cooperation criterion* and *biodiversity effect* to assess if *P. putida* biofilms promote or impede the attachment and biofilm formation by *S.* Java. Hereto, an in vitro model was developed and validated to simulate biofilm formation on the inside of the DWS of broiler chickens that approaches environmental conditions as close as possible. The effect of several *P. putida* field strains was evaluated against a *S.* Java strain isolated from broiler chicken drinking water.

## Results

### Validation of the in vitro biofilm model

To guarantee the relevance of the obtained results, a novel in vitro biofilm model was developed that closely resembles the environmental conditions of the DWS in broiler houses. Environmental conditions were simulated by growing biofilms on coupons made out of plastic drinking water lines for broilers. Low flow conditions in the DWS were simulated by shaking at a low speed (50 rpm) and an incubation temperature of 25 °C was chosen to mimic the average stable environmental temperature. Nutrient conditions were approached by applying a poor growth medium during biofilm formation. Finally, as specified in ‘Experimental procedures: Strain selection and preparation’, field strains (2 *Salmonella* strains; 3 *Pseudomonas putida* strains) previously collected from water, broiler feed and from inside surfaces of the DWS for broilers were used to simulate biofilm formation at this specific niche [41].

Before interactions between *P. putida* and *Salmonella* were investigated, mono-species biofilms were evaluated for the validation and implementation of the newly developed in vitro biofilm model. Biofilms were grown using a 6 log CFU/mL inoculum suspension of *S.* Java strain S1 (three independent times (days) with six technical replicates per time) and a 6 log CFU/mL inoculum suspension of *P. putida* P2 (three independent times (days) with five technical replicates per time). Only small standard deviations between replicates and no significant differences between independent times were observed for enumerations of biofilm experiments using the same strain (*p* = 0.0600 for S1 and *p* = 0.1738 for P2). An additional file shows this in more detail (see Additional file 1). Yet, OD measurements after crystal violet biomass staining provided large standard deviations between replicates and significant differences between times for both strains (*p* = 0.0009 for S1 and *p* = 0.0087 for P2). These results demonstrate the repeatability and reproducibility of the model for biofilm quantification based on CFU enumerations. Biofilm formation in the following experiments will therefore be evaluated based on bacterial counts.

### Influence of strain and inoculum density on mono-species biofilm formation

**Fig. 1:**
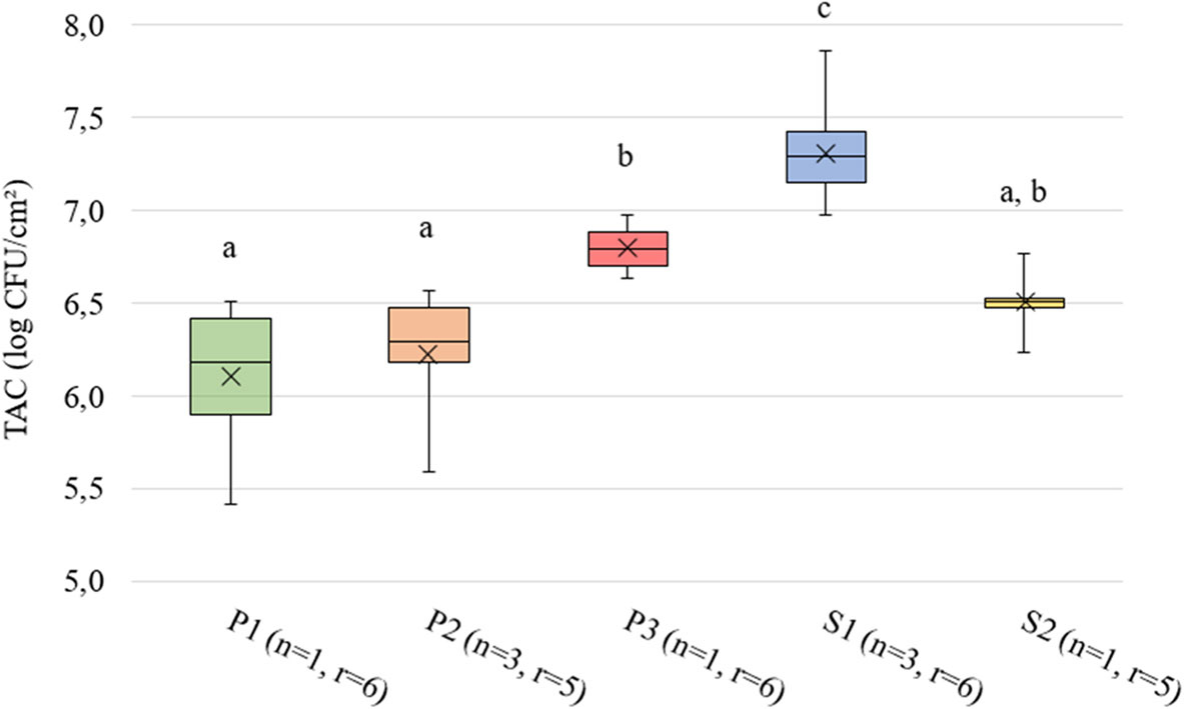
Mono-species biofilm formation by different field strains. The same inoculum density (6 log CFU/mL) was used for attachment of the different strains followed by biofilm quantification by enumerations of total aerobic counts (TAC, log CFU/cm^2^). Strains that did not show significantly different biofilm quantities are indicated with the same alphabetical character. *P*-values of ≤0.05 were considered as significant. The number of independent tests and technical replicates per test per strain is respectively indicated by n and r

**Fig. 2:**
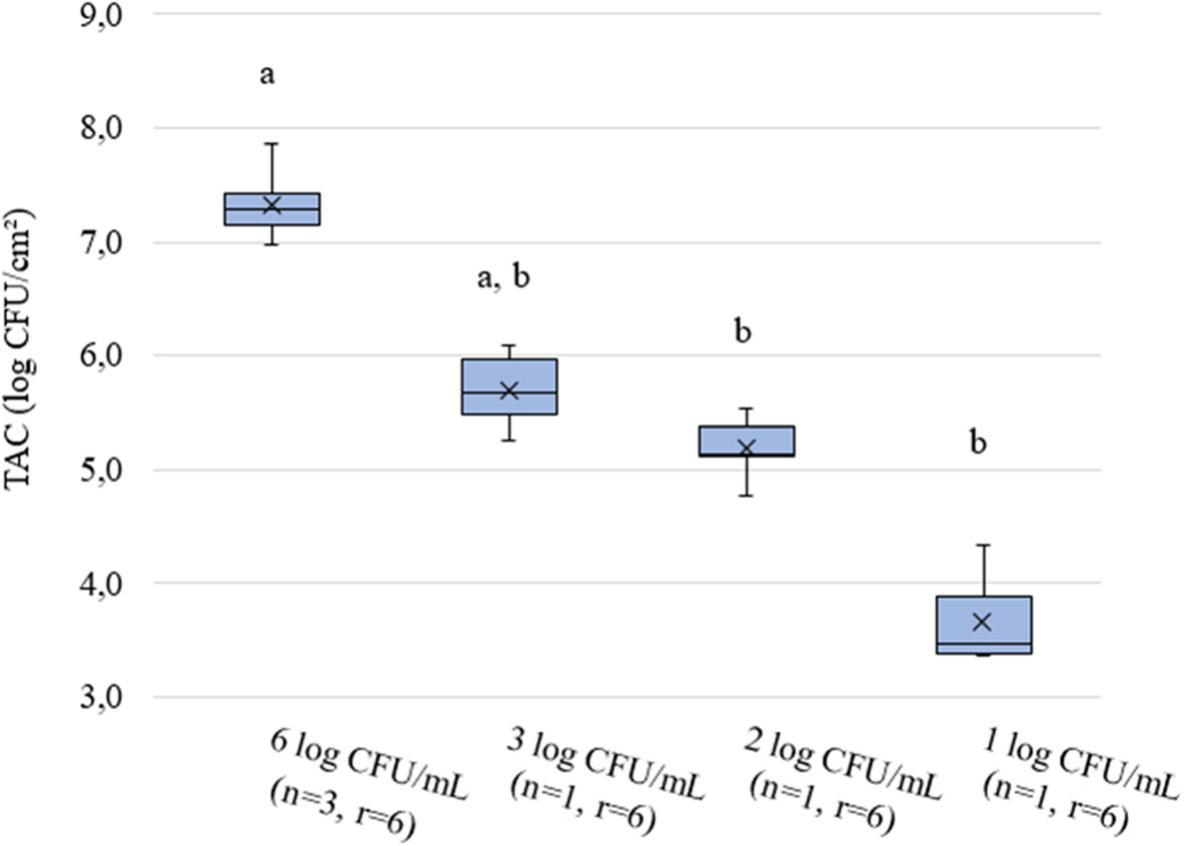
Mono-species biofilm formation by *S.* Java strain S1. Four different inoculum densities (6, 3, 2 and 1 log CFU/mL) were used for attachment followed by biofilm quantification by enumerations of total aerobic counts (TAC, log CFU/cm^2^). Inoculum suspensions that did not provide significantly different biofilm quantities are indicated with the same alphabetical character. *P*-values of ≤0.05 were considered as significant. The number of independent tests and technical replicates per test per strain is respectively indicated by n and r

The mono-species biofilm set-up was then used to study differences in biofilm-forming capacity of different *Salmonella* and *P. putida* strains isolated from broiler DWS and feed and to evaluate the influence of inoculum density on biofilm formation. This assay revealed significant differences in biofilm formation between different strains applied with the same inoculum density (Fig. 1) and between different inoculum densities of the same strain (Fig. 2). The application of 6 log CFU/mL of *P. putida* strains P1, P2 and P3 and *Salmonella* strains S1 and S2 resulted in biofilms of 6.10 ± 0.42, 6.22 ± 0.32, 6.80 ± 0.13, 7.31 ± 0.22 and 6.50 ± 0.19 log CFU/cm^2^, respectively. The different *P. putida* strains showed a similar trend in biofilm formation as in our previous study measuring biofilm biomass by crystal violet staining [41], with P1 producing the lowest amount of biofilm and P3 the highest. As *S.* Java (S1) has a higher biofilm-forming potential and is more prevalent and persistent in broiler practice compared to *Salmonella* Mbandaka (S2) ([64]; M. Cargnel, personal communication, Sciensano, April 26, 2019), S1 was selected to evaluate the influence of inoculum density on biofilm formation and to study the interactions with *P. putida*. Decreasing the inoculum density of *S.* Java S1 led to decreased biofilm formation. *S.* Java S1 applied with an inoculum density of 6 log CFU/mL thus provided the highest amount of biofilm of all strains based on microbial enumerations.

### Influence of strain on interaction and biocontrol effect

**Fig. 3:**
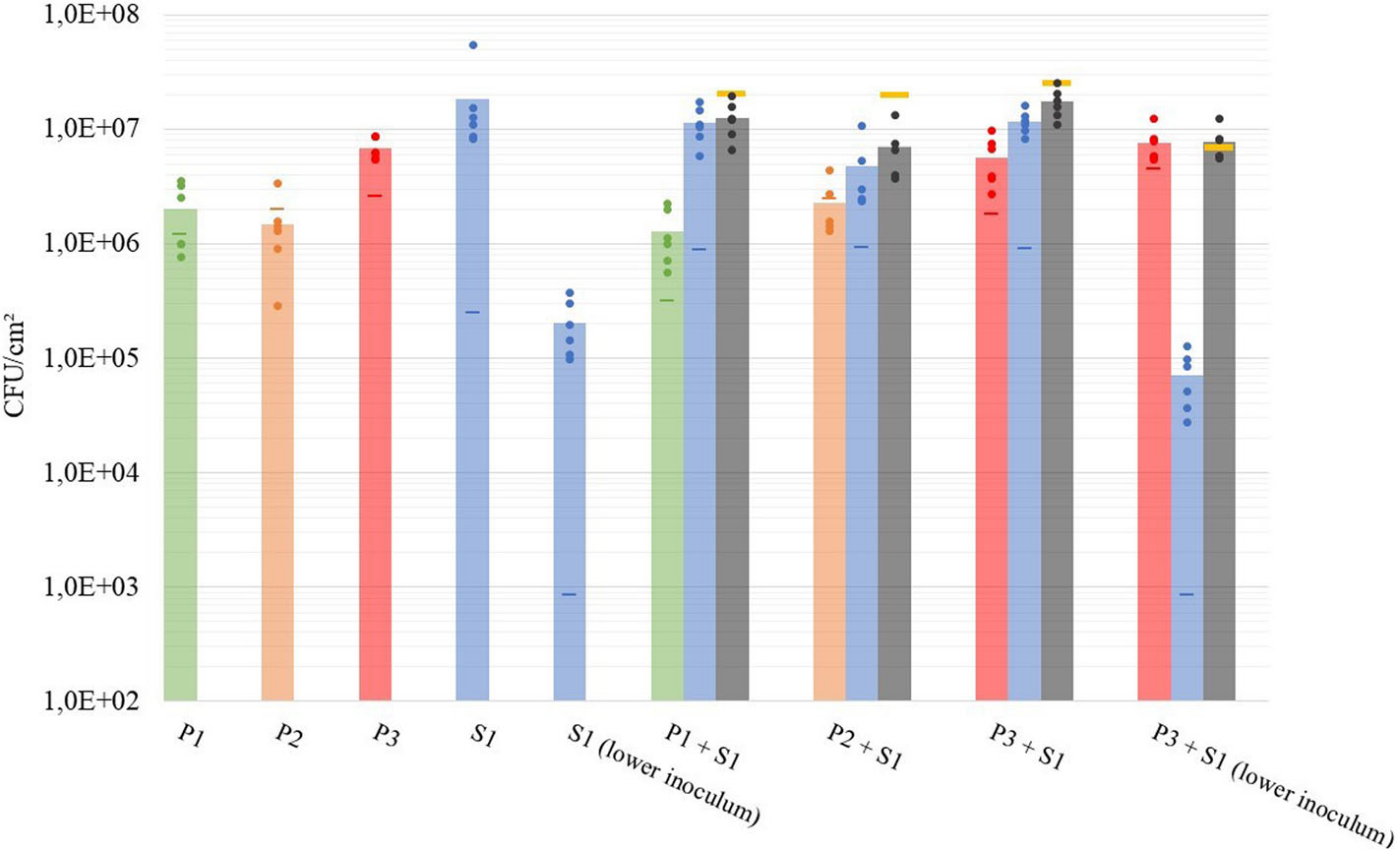
Influence of strain on the interaction and biocontrol effect. Bacterial counts of each strain (P1, P2, P3 and S1) in mono-species biofilms and in dual-species biofilms are indicated. Also the influence of S1 density on its bacterial counts in dual-species biofilms was examined. The results (in CFU/cm^2^) of six^*^ technical replicates (dots) and their average (columns) are shown per strain and in total (grey) for the dual-species biofilms. The actual inoculum density (CFU/ml) applied in every biofilm experiment is indicated with a horizontal line in the colour corresponding to the used strain. The total amount of cells expected for cooperation in dual-species biofilms is indicated as yellow horizontal lines. ^*^Dual-species biofilms consisting of P2 + S1 demonstrated one significant outlier, providing only five technical replicates in these results

To study the interaction between the *P. putida* strains and *S.* Java, dual-species biofilms were set up by simultaneously inoculating both species in a 1:1 ratio. The *cooperation criterion* states that cooperation only occurs if both strains show a lower cell number in monoculture than in mixed culture. The minimal requirement for cooperation thus entails that the total number of cells in dual culture is higher than the sum of the monocultures; otherwise the interactions are competitive, neutral or accidental [43]. Figure 3 shows that all three *P. putida* strains reduced the cell number of *S.* Java S1 cells in the dual-species compared to mono-species biofilms, indicating competitive interactions between both species. *P. putida* strain P2 mediated the largest reduction of *S.* Java S1. Biofilm formation by *S.* Java S1 in the presence of *P. putida* P2 was significantly lower (*p* = 0.0142) than in the presence of P1 or P3. The increase in cell number of P2 in dual-species biofilms is consistent with an exploitation of S1 by P2, resulting in dual-species biofilms with 6.31 ± 0.23 P2 and 6.60 ± 0.28 log CFU/cm^2^ S1. In contrast, the biofilms of other strain combinations (P1/S1; P3/S1) are characterized by mutual inhibition, with a decrease in cell numbers of both strains in the dual- compared to mono-species conditions. This resulted in dual-species biofilms with 6.05 ± 0.24 and 7.03 ± 0.17 log CFU/cm^2^ (P1/S1) or 6.72 ± 0.21 and 7.05 ± 0.10 log CFU/cm^2^ (P3/S1). Despite the inhibitory effect of *P. putida*, *Salmonella* was nevertheless still the dominant species in the dual-species biofilms when an equal amount of each strain was applied.

**Fig. 4:**
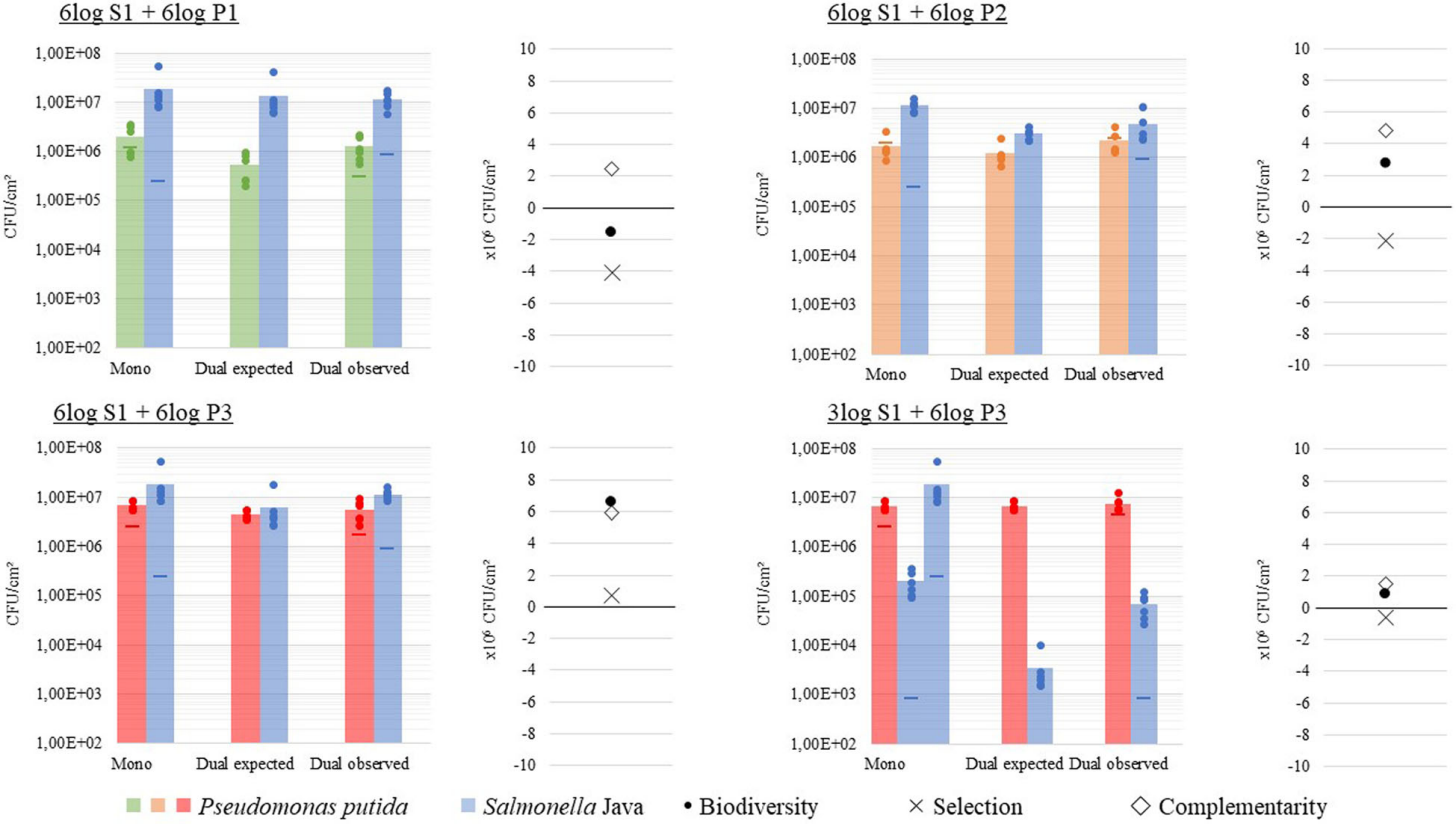
Study of the interaction between *Pseudomonas putida* and *S.* Java. Mono-species growth (Mono), expected dual-species growth (Dual expected) and observed dual-species growth (Dual observed) of *S.* Java S1 and *Pseudomonas putida* strains P1, P2 and P3, together with biodiversity, selection and complementarity effect of four dual-species biofilm experiments each with six^*^ technical replicates (dots) and their average (columns). The actual inoculum density (CFU/ml) applied in every biofilm experiment is indicated with a horizontal line in the colour corresponding to the used strain. ^*^Dual-species biofilms consisting of P2 + S1 demonstrated one significant outlier, providing only five technical replicates in these results

To further characterize the level and nature of competition we calculated the *biodiversity effect* [38, 49]. In case inter-species competition is equal to intra-species competition, the observed biofilm formation in dual-species conditions is expected to be equal to the mono-species biofilm formation, weighed by the inoculum densities (expected biofilm formation). As detailed in ‘Experimental procedures’, the *biodiversity effect* is defined as the difference between the observed (Y_O_) and this expected (Y_E_) dual-species biofilm formation and is therefore a measure for the extent to which inter-species interactions differ from intra-species interactions. Dual-species biofilms between S1 and P1 showed lower overall bacterial counts than expected (2.31E+ 06 CFU/cm^2^ less than expected for S1 and 7.47E+ 05 CFU/cm^2^ more than expected for P1), which is reflected in a negative *biodiversity effect*. On the other hand, culturing S1 together with P2 or P3 produced higher cell counts than expected as indicated by a positive *biodiversity effect* (Fig. 4). Indeed, in the P2/S1 biofilms, observed biofilm formation (Y_O_) was higher than expected based on intra-species competition (Y_E_), both for S1 and P2 (respectively 1.72E+ 06 and 1.03E+ 06 CFU/cm^2^). In the P3/S1 biofilms there was also an increase (observed vs. expected) in biofilm formation for both S1 (5.42E+ 06 CFU/cm^2^) and P3 (1.18E+ 06 CFU/cm^2^). In conclusion, P1/S1 biofilm formation appears to be lower than expected based on intra-species competition, whereas the opposite is the case for the P2/S1 and P3/S1 biofilms.

Since the *biodiversity effect* is the sum of the complementarity effect and the selection effect, both components were also analysed separately (Fig. 4). The negative *biodiversity effect* in dual-species biofilms of P1/S1 can be subdivided into a positive complementarity effect of 2.46E+ 06 and a negative selection effect of − 4.02E+ 06. In contrast, the complementarity and selection effect for P2/S1 biofilms were calculated as 4.87E+ 06 and − 2.12E+ 06, summing up to a positive *biodiversity effect*. For P3/S1 biofilms, the complementarity and selection effect were both positive (5.91E+ 06 and 6.98E+ 05 respectively). The positive complementarity effect in all three communities signifies that the intra-species competition among the bacteria of the same species is stronger than the inter-species competition between the different species. These results indicate that none of the three *P. putida* strains shows a complete overlap with *S*. Java in use of nutrients and space. Both species appear to populate partially separated ecological niches, relaxing the competition between them.

The positive selection effect in the P3/S1 biofilms indicates that S1, which is the best biofilm former in monoculture, shows the highest increase in relative biofilm formation in the dual culture biofilm compared to expected (RY_O_ – RY_E_ is 0.29 for S1 vs. 0.17 for P3). The negative selection effect in P1/S1 and P2/S1 biofilms on the contrary indicates that in these populations *P. putida* P1 and P2, which are worse biofilm formers in monoculture than S1, show the highest increase in relative biofilm formation (P1/S1: RY_O_ – RY_E_ is − 0.13 for S1 vs. 0.37 for P1; P2/S1: RY_O_ – RY_E_ is 0.15 for S1 vs. 0.60 for P2) [38, 49]. However, as in absolute terms P1 and P2 make still significant less biofilm in the dual culture than S1, this ‘ecological selection effect’ does not translate into evolutionary selection.

### Influence of pathogen inoculum density on interaction and biocontrol effect

Although *Salmonella* was inhibited by all three *P. putida* strains, it still dominated the dual-species biofilms if equal amounts of each species were applied. We therefore studied the influence of the pathogen’s inoculum density on the interaction with *P. putida* by lowering the inoculum density of S1 from 6 log CFU/mL to a more realistic 3 log CFU/mL [5]. *P. putida* P3 was selected for this study because of its high mono- and dual culture level of biofilm formation, as high cell numbers and enhanced biofilm formation offer advantages in terms of persistence [7, 58, 69]. In case of this lower inoculum, *Salmonella* S1 was repressed by P3 to a higher proportional extent than observed for the higher inoculum (Fig. 3). Interestingly, lowering the inoculum density of S1, changed the mutually competitive interaction (at 6 log CFU/mL inoculum) into an exploitative interaction (at 3 log CFU/mL inoculum), with slightly increased cell number of *P. putida* in dual-species compared to mono-species biofilms. This resulted in dual-species biofilms dominated by *P. putida,* with 6.86 ± 0.14 log CFU/cm^2^ of P3 and 4.79 ± 0.26 log CFU/cm^2^ of S1. The relative biofilm formation of both strains was again higher than expected (with 0.0037 for S1 and 0.11 for P3). The interaction was thus also still characterized by a positive complementarity effect (1.49E+ 06), pointing towards niche separation. However the selection effect turned negative (− 6.45E+ 05), indicating that the strongest increase in relative biofilm formation was made by P3, which is the worst biofilm former in monoculture (Fig. 4). The negative selection effect could not completely compensate for the positive complementary, leading to an overall positive *biodiversity effect*.

### Influence of sequential application of BCA and pathogen on interaction and biocontrol effect

**Fig. 5:**
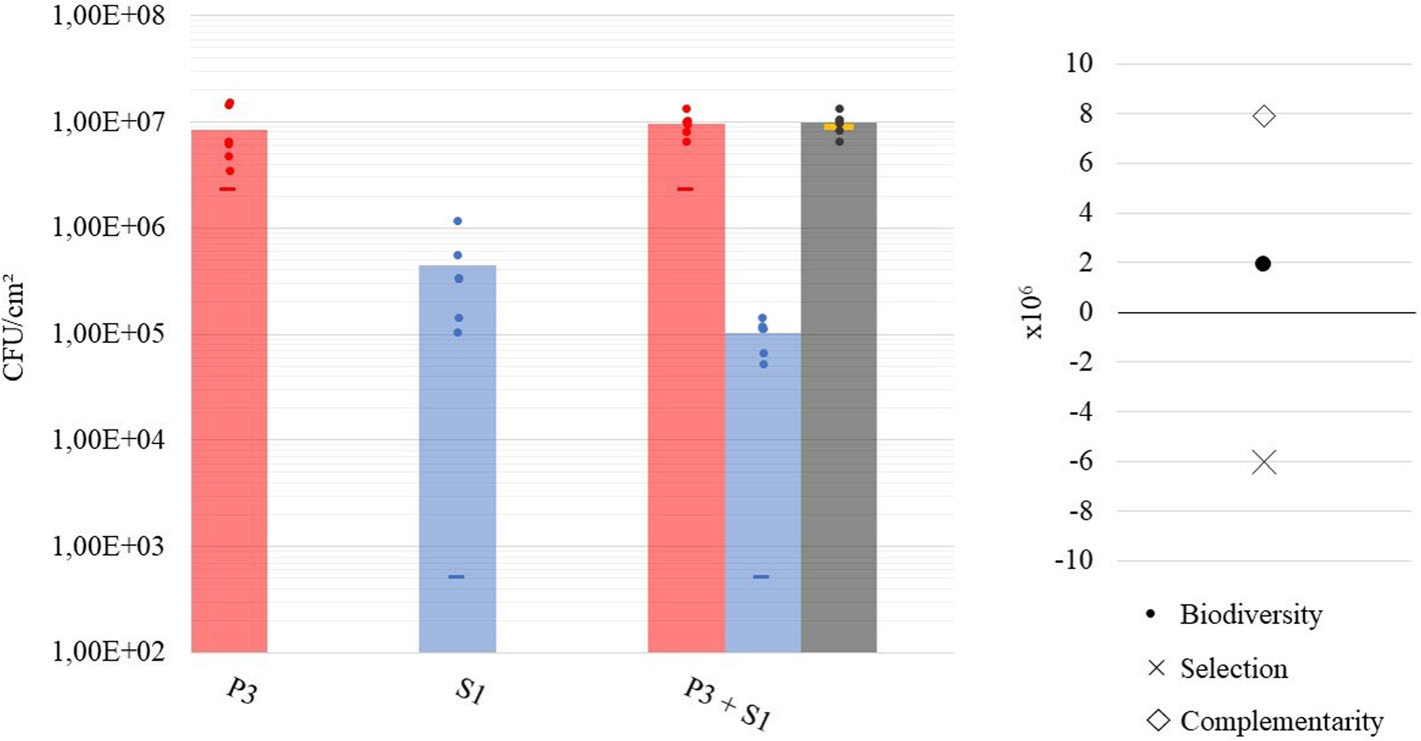
Influence of sequential application of biocontrol agent and pathogen on interaction and biocontrol effect. Cell number of strains P3 and S1 in mono-species biofilms and in dual-species biofilms are indicated. Results (in CFU/cm^2^) of the six technical replicates (dots) and their average (columns) are shown per strain and in total (grey) for the dual-species biofilms after 44 h of incubation, whereby S1 was only applied during the last 22 h. The actual inoculum density (CFU/ml) applied in every biofilm experiment is indicated with a horizontal line in the colour corresponding to the used strain. The total amount of cells expected for cooperation in dual-species biofilms is indicated as a yellow horizontal line. Also biodiversity, selection and complementarity effect, calculated based on the last 22 h of incubation i.e. when both strains were present, are shown

Biocontrol agents have potential to be applied in a preventive manner. We therefore finally studied the effect of sequential application of *P. putida* P3 and *S.* Java S1. The potential BCA strain P3 was first allowed to attach for 4 h and form a biofilm for 18 h, resulting in biofilms of 6.73 ± 0.13 log CFU/cm^2^. Then, an inoculum density of 3 log CFU/mL S1 was applied for attachment (4 h) and biofilm formation (18 h) on the pre-existing P3 biofilm. This resulted in dual-species biofilms dominated by *P. putida*, with 6.89 ± 0.11 log CFU/cm^2^ P3 and 4.98 ± 0.17 log CFU/cm^2^ S1 (Fig. 5). Absolute cell numbers of S1 in the resulting dual-species biofilms were lower than in mono-species S1 biofilms of 22 h old. *S.* Java biofilm formation on a pre-existing *P. putida* biofilm was thus significantly lower (*p* = 0.0039) than on clean surfaces. Also, the presence of a pre-existing biofilm of P3 could reduce the cell number of S1 (applied at 3 log CFU/mL) to a higher extent than when the strains were applied simultaneously, altough this further decrease in *Salmonella* level was not significant (*p* = 0.14). For P3, absolute cell numbers observed after 44 h of biofilm formation were higher in dual- compared to mono-species biofilms of 44 h. When only the growth during the last 22 h of the experiment is considered, absolute cell numbers for P3 also increased in dual- compared to mono-species biofilms. These results thus indicate that exploitative competition is also present between P3 and S1 in the sequential set-up. The *biodiversity effect* in this sequential biofilm experiment was then studied based on the last 22 h of incubation as a reference period, i.e. when both strains were present. The cell number of P3 in the pre-existing 22 h old biofilm was used to determine the P3 inoculum proportion (RY_E, P3_) in the *biodiversity effect* calculations. Both strains performed better than expected in the sequential set-up, with an increase in relative biofilm formation of 0.0054 for S1 and 0.76 for P3. Similarly to the simultaneous inoculation, the complementarity effect was positive (7.94E+ 06), pointing at niche separation, and the selection effect negative (− 6.02E+ 06), consistent with the highest increase in relative biofilm formation being made by the worst biofilm former in monoculture, which is P3. The negative selection effect could not compensate for the degree of complementarity, leading to an overall positive *biodiversity effect* (Fig. 5).

## Discussion

In recent years, biocontrol as an alternative for chemical disinfection has gained strong interest. Studies have already been performed in primary animal and plant production, food industry and even in hospitals [8, 28, 39, 65, 72, 74]. However, the possibility to use biocontrol agents against persistent pathogenic strains in the broiler environment has received only limited attention [2]. In this research, a realistic in vitro model for biofilm formation on the inside of the DWS was developed and validated. This model was utilized to study interactions between *Salmonella* Java and *Pseudomonas putida* strains previously isolated from DWS and to evaluate the potential of *P. putida* as BCA in this niche. Biofilm formation was evaluated based on bacterial counts. This quantification method proved more repeatable and reproducible compared to OD measurement of crystal violet after resolubilization. The high variation of the OD measurements for biofilm quantification was possibly due to the rinsing step under running tap water to remove the excess stain. During this step, an uncontrollable mechanic force is applied to the coupons whereby pieces of the biofilm can detach. Therefore, further optimisation is required to use OD measurement as a method to quantify biofilm formation in the newly developed in vitro biofilm model. However, bacterial counts are more valuable than OD measurements for mixed cultures as it allows to quantify the strains separately and determine the underlying social interactions. The developed model could be further expanded by incorporating a continuous flow. However, this approach would likely limit the throughput of the system.

The difference in monoculture biofilm-forming capacity between the three *P. putida* strains in the DWS in vitro model confirmed the previous observations concerning biofilm formation by these strains in 96-well MTPs [41]. Differences in biofilm-forming capacity between strains of the same species are commonly reported and can be due to mutations in biofilm regulating genes [1, 4, 12, 34, 37]. Very few literature was found concerning monoculture biofilm-forming capacity of *S.* Java. Agarwal et al. [1] screened a multitude of *Salmonella* serotypes, among which one *S.* Java strain, for biofilm formation in 96-well MTPs. The *S.* Java strain was evaluated as a weak biofilm former based on OD measurements. In the current study, where biofilm formation was evaluated under more realistic conditions, the *S.* Java field strain was evaluated as the best biofilm former based on bacterial counts compared to the other strains that were included (among which another *Salmonella* serotype i.e. *Salmonella* Mbandaka). Even at low inoculum densities, which are more realistic for the investigated niche [5], *S.* Java was capable to form a significant amount of biofilm. It was already demonstrated for *Listeria monocytogenes* that persistent strains show increased biofilm formation relative to non-persistent strains [7, 58, 69]. The strong biofilm-forming capacity of the *S.* Java strain in this study could therefore be an explanation for the persistent character of this *Salmonella* serotype in broiler houses. A framework based on the *cooperation criterion* [43] and *biodiversity effect* (consisting of a selection effect and a complementarity effect) [38, 49] was applied to characterize the social interactions between *S*. Java and the three *P. putida* strains. The study of social interaction provides essential information to identify effective BCAs. Several characteristics should be taken into account. First, it is advantageous if the BCA shows a strong inhibitory effect and a high cell number when co-cultured with the strain to be controlled. If the BCA has a higher number of cells than the unwanted strain this would indicate the BCA has a higher fitness in this niche, leading to gradual enrichment (selection) and higher dominance of the BCA over time. Furthermore, the niche overlap between the BCA and the unwanted strain should be maximal, preferentially leading to high levels of interference competition. Both factors are reflected in a low (preferentially negative) complementarity effect.

All evaluated *P. putida* strains were able to some extent to reduce the attachment and biofilm formation by *S.* Java, supporting the potential of *P. putida* as a BCA against *S.* Java in the DWS of broiler houses. The ability of *Pseudomonas* strains to inhibit the growth of several pathogenic bacteria, among which *Salmonella,* was previously attributed to the production of iron-capturing siderophores and the toxic pigment pyocyanin [11, 17, 24, 29, 45]. *P. putida* specifically also produces several biosurfactants that can inhibit biofilm formation and even break down existing biofilms [31]. In addition, in silico genome mining revealed two clusters for biosynthesis of bacteriocins and one cluster for a type I polyketide synthase. *P. putida* thus has a wide arsenal of weaponry that could inhibit *S*. Java biofilm formation. The positive complementarity in the co-cultures does not point towards strong interference competition among *S*. Java and *P. putida*. However, a positive complementarity does not exclude that interference competition takes place as it is possible that the inhibitory effect of interference competition is compensated by the benefits of niche separation, resulting in a net positive complementarity.

All three *P. putida* strains showed different inhibitory effects on *S.* Java. When inoculated in equal amounts, strain P2 inhibited *S.* Java to the highest extent and even increased its own biofilm formation compared to mono-culture. The other *P. putida* strains also inhibited *S*. Java significantly, albeit to a lower extent, and they formed less biofilm in mixed- than in mono-culture. The observation that all three *P. putida* strains engage in competitive interactions with *S*. Java fits with a growing body of recent theoretical and experimental work indicating that competition, not cooperation, dominates interactions among microbial species [22, 43, 49]. More specifically, interactions between *Pseudomonas aeruginosa* and *Salmonella* Enteritidis and Typhimurium, between *Pseudomonas fluorescens* and *Salmonella* Typhimurium, Montevideo and Poona, and between *P. putida* and *Salmonella enterica* were also identified as competitive ([13, 33, 46–48]). In general, interactions between *Pseudomonas* and other *Enterobacteriaceae* such as *Escherichia coli* and *Klebsiella pneumoniae* are also predominantly competitive, however the species dominating the co-culture and whether mutual inhibition or exploitation occurs is strongly strain- and condition-dependent ([10, 15, 23, 35, 71). Moreover, the type of interaction between strains is greatly dependent on environmental conditions, among which the stress gradient plays an important role [51]. Therefore, the presence of medication administered through the DWS can also influence the interaction between *P. putida* and *S*. Java in practice.

Although all interactions were competitive in nature, the positive complementarity effects in all strain combinations indicate that the niches between both species do not completely overlap, alleviating the competitive interactions. In addition, despite the inhibitory effect of *P. putida, Salmonella* remained the dominant species when equal inoculum densities were applied as counts for *Salmonella* were always higher than counts for *Pseudomonas* spp. The dominance of *Salmonella* Typhimurium relative to *Pseudomonas aeruginosa* in dual-species biofilms was already described by Pang et al. [47], but in the same study *Salmonella* Enteritidis was equally distributed to *P. aeruginosa*. In contrast to our study, coexistence between *Pseudomonas* and *Salmonella* Agona enhanced biofilm formation by *S.* Agona in terms of increased biovolume in the study of Habimana et al. [25]. Overall, this suggests that the behaviour of *Salmonella* in dual-species biofilms with *Pseudomonas* is strongly dependent on respectively serotype and strain. In addition, differences in biofilm growth conditions (flow, incubation time, incubation temperature, stress factors, surface type, etc.) could also lead to different interactions between the strains [16, 51].

Consistently, we found that changing the inoculum ratio affects the outcome of competition greatly. When the inoculum proportion of P3/S1 was lowered to a more realistic 1:0.001, P3 was able to exploit resources provided by competitor *S*. Java and dominate the biofilm. This exploitation could for example be due to superior positioning in the biofilm or the consumption of metabolic by-products generated by *Salmonella* [30, 49, 50, 55]. When *P. putida* was first allowed to form a biofilm on the surface of the DWS and only afterwards *S.* Java was applied, a further increase in the competitive effect against *S*. Java was established, as evident by a stronger percentage reduction in *Salmonella* cell numbers. Again, *P. putida* was able to dominate the biofilm and exploit *S*. Java. One possible explanation for the enhanced inhibitory effect in the sequential set-up is that *P. putida* covers the abiotic surface and prevents the adhesion of *Salmonella* in a process called surface blanketing [53]*.* However, surface blanketing is unlikely, due to the low density of the *P. putida* biofilms. Indeed, prior research indicates that at similar densities *Pseudomonas* forms a sparse biofilm that does not cover the complete surface [21]. The above described effects of nutrient and interference competition are therefore likely more important.

Although *S.* Java was inhibited by the *P. putida* strains, prevention of *Salmonella* colonization was far from complete. Moreover, it is not known which impact this reduction in *S*. Java biofilm formation will have on the prevalence of *S*. java in broilers or on broiler meat. Given the strain variations observed in this study, other *P. putida* strains might be able to reduce *S.* Java to an even higher extent. It would therefore be interesting to evaluate the biocontrol potential of additional *P. putida* strains, possibly in combination with other *Salmonella*-biocontrol species. Moreover, biofilms on the inside of the DWS in broiler houses are composed of a diverse range of microorganisms [41], which might also interact with pathogen and BCA. Future biocontrol assays should take this species diversity into account.

Another important factor to consider is that, although co-culturing *Salmonella* and *Pseudomonas* can lead to less biofilm formation by *Salmonella*, different studies reported an increased *Salmonella* tolerance to disinfectants in these mixed species biofilms [33, 48]. Parijs and Steenackers [49] reported this increased tolerance can be a consequence of competitive release in the biofilm upon treatment or of an increase in inherent tolerance due to the presence of competing species. Another downside of biofilms present on the inside of the DWS in broiler houses (independent on the strain composition) is clogging of the pipes and capture of medicine particles, leading to under dosing of the animals and increasing the risk for animal health and the development of drug resistant strains [27, 54]. Therefore also the combination of biocontrol strategies and chemical (disinfection/drug) treatments should be investigated.

## Conclusions

In conclusion, the present study indicates the potential of *P. putida* as a biocontrol agent against *S.* Java. Competitive interactions were observed between both genera in a newly developed and validated in vitro model that simulates biofilm formation on the inside of the DWS in broiler houses under realistic conditions. When equal inocula were simultaneously applied, the interaction between *S*. Java and *P. putida* strains P1 and P3 was characterized as mutually inhibitory, whereas *P. putida* strain P2 showed an exploitation of *S*. Java. Lowering the inoculum density of *S*. Java changed the mutually competitive interaction with *P. putida* strain P3 also into an exploitation by P3 and enhanced the competitive inhibition of *S*. Java. A further increase in *S*. Java inhibition was established by allowing *P. putida* (strain P3) to form a mature biofilm before applying *S.* Java. Future studies should extend this work by including more complex resident microbial communities from the DWS niche, additional *Salmonella* strains and other zoonotic pathogens frequently occurring in the broiler industry (such as *Campylobacter* spp.), as well as chemicals typically applied to clean and disinfect the systems.

## Methods

### Strain selection and preparation

To study the potential of *Pseudomonas putida* as BCA against *Salmonella*, several field strains from broiler houses were used (Table 1). The *P. putida* strains were in a previous study [41] classified as weak (P1), moderate (P2) and strong (P3) biofilm formers in 96-well microtiter plates (MTPs). This classification was based on the absorbance measured at 590 nm after crystal violet staining of the biofilms, which was divided into groups according to Stepanović et al. [61].

**Table 1:** Field strains to study the potential of *Pseudomonas putida* as BCA against *Salmonella* Java

Strain	Identity	Abbreviations	Origin
KS243	*Salmonella enterica subsp. enterica* serovar Paratyphi B variant Java	*Salmonella* Java, *S*. Java, S1	Drinking water of broiler chickens on Belgian broiler farm
MB1560	*Salmonella enterica* subsp. *enterica* serotype Mbandaka	*Salmonella* Mbandaka, S2	Broiler feed
MB6188	*Pseudomonas putida*	P1	Inside surfaces of the DWS in broiler houses
MB6189	*Pseudomonas putida*	P2	Inside surfaces of the DWS in broiler houses
MB6275	*Pseudomonas putida*	P3	Inside surfaces of the DWS in broiler houses

For the preparation of the bacterial suspension for inoculation in the in vitro biofilm model, strains were streaked on Plate Count Agar (PCA, Oxoid, CM0325, Basingstoke, Hampshire, England) from their glycerol stocks at − 80 °C and incubated for 24 h at 37 °C for *Salmonella* strains and 48 h at 30 °C for *Pseudomonas* strains. Subsequently, one colony from PCA was transferred to a test tube containing 10 mL of Tryptone Soya Broth (TSB, Oxoid, CM0129). An overnight culture was obtained by incubating the broth for 18 h at 30 °C for *Pseudomonas* (8 log CFU/mL) or 18 h at 37 °C for *Salmonella* (9 log CFU/mL) strains. Quantification of the overnight culture was done by plating on PCA and incubation for 72 h at 30 or 37 °C depending on the species. Finally, overnight cultures were diluted in sterile ¼ Ringer’s solution (Biokar, BR00108, Beauvais, France) to the desired density (3 or 6 log CFU/mL depending on the corresponding biofilm set-up) and the resulting suspension is called the inoculum suspension. Actual inoculum densities were calculated based on the CFU quantification of the overnight cultures and were taken into account in the study of the interactions between strains in dual-species biofilms.

### Model preparation

Coupons, 35x10x2mm, were cut from new plastic drinking water lines commonly used in broiler houses (Swii’Flo, Roxell, Maldegem, Belgium). Before use, these coupons were sterilized in 70% ethanol for 10 min and dried in a laminar flow cabinet. The sterilized coupons were vertically placed in the wells of a 6-well MTP (Novolab, SPL30006, Geraardsbergen, Belgium) using sterilized tweezers in a way that only the 2 mm sides of the coupons touch the wells.

### Mono- and dual-species biofilm formation

#### Attachment of the bacterial strains

For each independent test (n) of each tested strain (combination) and condition, a 6-well MTP was used for biofilm formation. In this plate, 11 mL of inoculum suspension was added per well (technical replicates = r) to completely submerge the coupons. A second 6-well MTP was used as blank control. In this plate, 11 mL of diluted TSB (equally diluted as the inoculum suspension) was applied in three wells. Only for the validation of the model, a third MTP was used for biofilm formation. Well plates were incubated in an incubator with shaker (Adolf Kuhner ag, LT-V 89799.89, Basel, Switzerland) for 4 h at 25 °C and 50 rpm making attachment of the bacteria to the coupons possible. After incubation, coupons were removed from the 6-well MTPs and transferred to 15 mL falcon tubes (Sigma-Aldrich, Z720461-50EA, Overijse, Belgium) using sterilized tweezers. To remove non-attached bacteria, coupons were rinsed once by submerging them in 10 mL sterile ¼ Ringer’s solution in the falcon tubes. Afterwards, the ¼ Ringer’s suspension was discarded and coupons with attached bacteria were placed vertically in new 6-well MTPs.

#### Biofilm formation by the attached bacterial strains

The new MTP’s with coupons were filled with 11 mL of sterile 1/20 diluted TSB per well and subsequently incubated for 18 h at 25 °C and 50 rpm to allow the attached bacteria to form biofilm. After incubation, coupons were removed from the 6-well MTPs and transferred to 15 mL falcon tubes using sterilized tweezers. To remove non-attached bacteria, coupons were rinsed three times by consecutively adding 10 mL of sterile ¼ Ringer’s solution in the falcon tubes and discarding the suspension. Finally, coupons were transferred to new sterile 15 mL falcon tubes.

### Quantification of biofilm formation based on bacterial counts

Coupons originating from the first MTP were used for quantification of biofilm formation by conventional microbial enumeration methods. Three blank control coupons from the second MTP were also counted to ensure no contamination did occur during analysis. First, 10 mL of sterile ¼ Ringer’s solution was added to the falcon tubes containing the coupons. Then, three consecutive rounds of sonication for 30s at 42 kHz in a ultrasonic water bath (Branson, 2510, Eemnes, The Netherlands) and vortexing for 30s were performed to harvest the biofilm.

The liquid suspension containing the detached biofilm cells was plated on Tryptone Soya Agar (TSA, Oxoid, CM0131) for enumerations of total aerobic count (TAC) and a second, more selective, medium. This selective medium was Xylose Lysine Desoxycholate Agar (XLD, Oxoid, CM0469) for *Salmonella* and *Pseudomonas* Agar Base (PAB; Oxoid, CM0559) with *Pseudomonas* CFC Selective Agar Supplement (Oxoid, SR0103) for *Pseudomonas.* Appropriate 10-fold dilutions were made in sterile 0,1% w/v Peptone Water with 0,85% w/v Salt (BioTrading, K110B009AA, Mijdrecht, The Netherlands) and pour plated. TSA plates were incubated for 72 h at 30 °C or 37 °C for *Pseudomonas* or *Salmonella* biofilms, respectively. XLD plates were incubated for 72 h at 37 °C and PAB plates were incubated for 72 h at 30 °C. The limit of quantification (LOQ) for microbiological enumerations was 1,16 log CFU/cm^2^.

### Quantification of biofilm formation based on biomass

For the validation of the model, six coupons from the third MTP used for biofilm formation and three blank control coupons from the second MTP were used for quantification of biofilm formation based on biomass. 10 mL of a 0.1% crystal violet solution (containing 0.1 g/100 mL crystal violet (Merck, 101,418, Darmstadt, Germany) dissolved in one part of methanol (Biosolve, 13,687,802, CE Valkenswaard, The Netherlands), one part of isopropanol (Merck, 1.09634) and 18 parts of Phosphate Buffered Saline (Oxoid, BR0014G)) was added to each of the falcon tubes for 20 min and shaken (Fisher Bioblock Scientific, KL2 6118 CU 00246, Merelbeke, Belgium) at 350 rpm for the staining of the total biomass of the biofilm on the coupons. The excess stain was removed by placing the tubes under gently running tap water. Retained crystal violet was dissolved by adding 10 mL of 33% acetic acid (Merck, 1.00063) for 15 min at 350rmp. The absorbance was measured at 590 nm using a spectrophotometer (Jasco, V-660, Pfungstadt, Germany). OD-measurements of the blank control coupons were subtracted from the OD-measurements of the biofilm coupons.

### Study of interactions between bacterial strains in dual-species biofilms

In this study, the *cooperation criterion* and the *biodiversity effect* were calculated to determine social interactions between *S.* Java and *P. putida* and to consequently assess the potential of *P. putida* as BCA. The *cooperation criterion* requires that the inoculation density in co-culture equals the sum of inoculation densities of the monocultures whereas the *biodiversity effect* imposes that the inoculation density of each species in co-culture should be its inoculation density in monoculture divided by the number of species in co-culture [49]. A preliminary experiment was conducted growing mono-species biofilms of *S*. Java in both set-ups but no differences in final biofilm growth were observed. Therefore, both the *cooperation criterion* and the *biodiversity effect* were calculated based on the results of dual-species biofilms where the inoculation density equals the sum of the inoculation densities of the monocultures.

Concerning the *cooperation criterion*, counts for TAC of the dual culture were compared with the sum of the counts for TAC of the two monocultures of *Salmonella* and *Pseudomonas*. Also, counts for *Salmonella* spp. on XLD and *Pseudomonas* spp. on PAB were compared between mono and dual cultures.

The *biodiversity effect* can be calculated as follows [38]:

$$ \Delta  Y={Y}_O-{Y}_E=N\overline{\Delta  RY}\ \overline{M}+ Ncov\left(\Delta  RY,M\right) $$

N = number of species in mixed-species community.

*M*_*i*_= growth of species i in mono-species conditions.

*RY*_*E*, *i*_= expected relative biofilm growth of species i in mixed-species conditions, which is its proportion inoculated.

*RY*_*O*, *i*_ = *Y*_*O*, *i*_/*M*_*i*_= observed relative growth of species i in mixed-species.

*∆RY*_*i*_ = *RY*_*O*, *i*_ − *RY*_*E*, *i*_ = deviation from expected relative growth of species i in mixed-species conditions.

This biodiversity effect measures how inter-species interactions differ from intra-species interactions based on the difference between the observed multi-species biofilm productivity and an expected value derived from the productivity in mono-species biofilms. The biodiversity effect is the sum of the selection effect (*Ncov*(*∆RY*, *M*)) and the complementarity effect $\left(N\overline{\Delta  RY}\ \overline{M}\right)$. The selection effect comprises deviations from the expected productivity due to relative enrichment of strong biofilm or weak biofilm formers. A positive selection effect indicates enrichment of the strongest monoculture biofilm formers, whereas a negative selection indicates that the weaker biofilm producers are enriched. The complementarity effect measures to what extent deviations from the expected relative productivity are compensated by the other strains. It comprises all deviations from the expected productivity not explained by the selection effect. A positive complementary indicates some degree of niche separation between the different strains whereas a negative complementary points towards interference competition. Interpretation of these effects is further explained in the results and discussion section.

### Statistical analysis

Statistical analyses on the obtained microbiological and biomass results were carried out using Statistical Analysis System software (SAS®, version 9.4, SAS Institute Inc., Cary, NC, USA). First, normal distribution of the OD measurements and of the log transformed enumerations per microbiological parameter per biofilm experiment were evaluated based on the histogram and QQ plot. For the evaluation of the reproducibility of the model system, a Kruskal Wallis test was used to compare results for OD measurement and enumerations between the three experiments per strain. For the comparison of mono-species biofilm formation of different bacterial strains, enumerations of TAC were evaluated per experiment using ANOVA. Post-hoc pairwise comparisons were made using Scheffe test. For the comparison of mono-species biofilm formation with different inoculum densities, enumerations of TAC were evaluated per experiment using a Kruskal Wallis test. Post-hoc pairwise comparisons were made using Dunn test. For the comparison of the quantification of different dual-species biofilms (with different strains, different inoculum densities or different application order) again a Kruskal Wallis test was performed on enumerations of TAC, *Pseudomonas* spp. and *Salmonella* spp. followed by a post-hoc pairwise comparison using Dunn test to indicate possible differences. *P*-values ≤0.05 were considered significant.

## Supplementary Information

**Additional file 1:.** Validation of the in vitro biofilm model.

## Data Availability

All data generated or analysed during this study are included in this published article and its supplementary information files.
