# Supplementary material for: Pseudomonas putida as a potential biocontrol agent against Salmonella Java biofilm formation in the drinking water system of broiler houses
Source: BMC Microbiol. 2020 Dec 11;20:373. doi: 10.1186/s12866-020-02046-5 (PMC7731557; doi:10.1186/s12866-020-02046-5)
Supplement: Supplementary file 1 — Additional file 1:. Validation of the in vitro biofilm model. [file 12866_2020_2046_MOESM1_ESM.docx]

**Additional file 1: Validation of the *in vitro* biofilm model**

Table 1: Evaluation of the repeatability and reproducibility of the biofilm model using S. java. The biofilm assay was performed on three independent times (days) with six technical replicates per time. Quantification of biofilm formation was performed by enumerations for total aerobic count (TAC) and Salmonella spp. (SALMONELLA) and quantification of the total biomass by OD measurement at 590nm (OD_590nm_).

| **Technical replicates** | **TAC (log CFU/cm²)** | | | **SALMONELLA (log CFU/cm²)** | | | **OD_590nm_** | | |
| --- | --- | --- | --- | --- | --- | --- | --- | --- | --- |
|  | **Day 1** | **Day 2** | **Day 3** | **Day 1** | **Day 2** | **Day 3** | **Day 1** | **Day 2** | **Day 3** |
| 1 | 7.10 | 7.54 | 7.34 | 7.10 | 7.52 | 7.26 | 0.1934 | 0.4289 | 0.0312 |
| 2 | 7.15 | 7.44 | 7.22 | 7.19 | 7.42 | 6.97 | 0.2719 | 0.3806 | 0.0364 |
| 3 | 6.98 | 7.35 | 7.26 | 6.93 | 7.32 | 6.97 | 0.1922 | 0.3587 | 0.0513 |
| 4 | 7.05 | 7.45 | 7.63 | 6.91 | 7.33 | 7.56 | 0.1398 | 0.1937 | 0.0196 |
| 5 | 7.15 | 7.31 | 7.16 | 7.04 | 7.10 | 6.99 | 0.1012 | 0.2514 | 0.0307 |
| 6 | 7.86 | 7.28 | 7.39 | 7.74 | 7.13 | 7.27 | 0.1259 | 0.2075 | 0.0303 |
| Average | 7.21 | 7.39 | 7.33 | 7.15 | 7.30 | 7.17 | 0.1707 | 0.3035 | 0.0333 |
| Standard deviation | 0.32 | 0.10 | 0.17 | 0.30 | 0.16 | 0.24 | 0.0617 | 0.0987 | 0.0104 |
| P value^1^ | 0.0600 | | | 0.2397 | | | 0.0009 | | |

^1^ P ≤ 0.05 was considered significant

Table 2: Evaluation of the repeatability and reproducibility of the biofilm model using P. putida. The biofilm assay was performed on three independent times (days) with five technical replicates per time. Quantification of biofilm formation was performed by enumerations for total aerobic count (TAC) and Pseudomonas spp. (PSEUDOMONAS) and quantification of the total biomass by OD measurement at 590nm (OD_590nm_).

| **Technical replicates** | **TAC (log CFU/cm²)** | | | **PSEUDOMONAS (log CFU/cm²)** | | | **OD_590nm_** | | |
| --- | --- | --- | --- | --- | --- | --- | --- | --- | --- |
|  | **Day 1** | **Day 2** | **Day 3** | **Day 1** | **Day 2** | **Day 3** | **Day 1** | **Day 2** | **Day 3** |
| 1 | 6.57 | 5.59 | 6.56 | 6.53 | 5.46 | 6.55 | 0.0038 | -0.7482 | 0.0596 |
| 2 | 6.30 | 5.63 | 6.45 | 6.20 | 5.15 | 6.40 | 0.2007 | -0.6769 | 0.1957 |
| 3 | 5.76 | 6.23 | 6.51 | 6.11 | 6.02 | 6.57 | 0.2654 | -0.3144 | 0.0363 |
| 4 | 6.29 | 6.49 | 6.21 | 6.15 | 6.48 | 6.25 | -0.0345 | -0.7476 | 0.0277 |
| 5 | 6.32 | 6.15 | 6.23 | 5.96 | 6.15 | 6.31 | -0.0268 | -0.7458 | 0.0827 |
| Average | 6.25 | 6.02 | 6.39 | 6.19 | 5.85 | 6.42 | 0.0817 | -0.6466 | 0.0804 |
| Standard deviation | 0.30 | 0.39 | 0.16 | 0.21 | 0.54 | 0.14 | 0.1408 | 0.1882 | 0.0679 |
| P value^1^ | 0.1738 | | | 0.0517 | | | 0.0087 | | |

^1^ P ≤ 0.05 was considered significant
